# Comparative One-Year Outcomes of T-Hook^®^-Versus Kahook Dual Blade^®^-Assisted Ab Interno Trabeculotomy Combined with Phacoemulsification for Primary Open-Angle Glaucoma

**DOI:** 10.3390/jcm15103834

**Published:** 2026-05-15

**Authors:** Yoshitaka Hoshino, Masatoshi Omi, Hidetsugu Mori, Masato Ishino, Tatsunori Kiriishi, Shimpei Oba, Hisanori Imai

**Affiliations:** Department of Ophthalmology, Kansai Medical University, Osaka 573-1010, Japanimai.his@kmu.ac.jp (H.I.)

**Keywords:** primary open-angle glaucoma (POAG), ab interno trabeculotomy, minimally invasive glaucoma surgery (MIGS), Kahook dual blade, T-hook, transient IOP spike

## Abstract

**Background:** The T-hook is a recently introduced device for ab interno trabeculotomy, first reported in 2022. This study compared the one-year surgical outcomes of Kahook Dual Blade (K group)- and T-hook (T group)-assisted trabeculotomy combined with phacoemulsification in patients with primary open-angle glaucoma (POAG). **Methods:** This retrospective study included patients with POAG who underwent 180° ab interno trabeculotomy combined with phacoemulsification at our institution between June 2018 and September 2024 and were followed for at least 12 months. Changes in intraocular pressure (IOP), mean IOP reduction rate, number of antiglaucoma medications, postoperative complications (hyphema and transient IOP spikes), and cumulative surgical success rates were evaluated. **Results**: A total of 45 patients (61 eyes) were included, comprising 29 patients (42 eyes) in the K group and 16 patients (19 eyes) in the T group. A transient increase in IOP at one week postoperatively observed in the K group (*p* < 0.0001); however, both groups demonstrated significant IOP reduction from baseline after 1 month (*p* < 0.05). The mean IOP reduction rate at 12 months did not differ significantly between groups (*p* = 0.0720, ANCOVA). The number of antiglaucoma medications significantly decreased at all postoperative time points in both groups compared with baseline (*p* < 0.05). Kaplan–Meier survival analysis revealed no significant difference in cumulative surgical success rates between groups (*p* = 0.6217). The incidence of hyphema was comparable between groups (*p* = 1.00), whereas transient IOP spikes occurred significantly more frequently in the K group (*p* = 0.0057). **Conclusions:** While both procedures demonstrated comparable intraocular pressure-lowering efficacy, T-hook-assisted trabeculotomy was associated with fewer transient postoperative IOP spikes during the early postoperative period in this cohort.

## 1. Introduction

Trabeculectomy has long been the standard surgical treatment for glaucoma for several decades [1]. However, in recent years, surgical strategies have shifted from ab externo procedures toward ab interno approaches, particularly minimally invasive glaucoma surgery (MIGS) [2]. Compared with conventional filtering surgery, ab interno procedures are associated with a more favorable safety profile and preserve the conjunctiva by accessing the trabecular meshwork from within the anterior chamber, thereby maintaining future surgical options [3].

A variety of MIGS devices have been developed and can be broadly classified according to their anatomical targets [4]. These include devices targeting the Schlemm’s canal or trabecular outflow pathway, such as the Kahook Dual Blade [5], iStent [6], Streamline [7], and Hydrus Microstent [8] devices targeting the suprachoroidal outflow pathway, such as the iStent Supra [9] and subconjunctival shunt procedures, including the PreserFlo MicroShunt [10].

**Figure 1:**
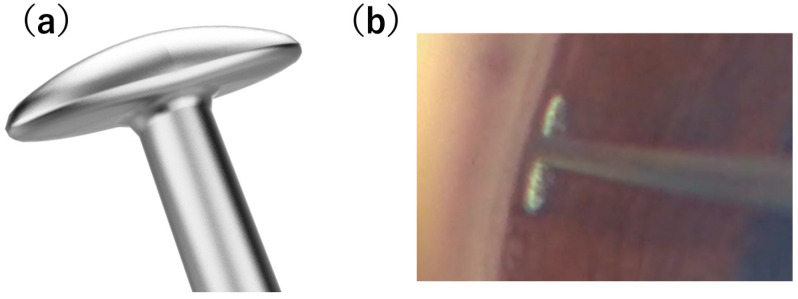
(**a**). T-hook device used for ab interno trabeculotomy. The image is reproduced from the manufacturer’s official website (Inami & Co., Ltd., Tokyo, Japan; https://inami.co.jp/products/m-2225.html, accessed on 10 May 2026) with permission or appropriate attribution. (**b**). Part of the intraoperative video (T-hook ab interno trabeculotomy).

The T-hook is a recently introduced device for ab interno trabeculotomy, first reported in 2022. Its T-shaped tip enables bidirectional incision of the trabecular meshwork, while its rounded design is intended to minimize damage to the outer wall of Schlemm’s canal [11] (Figure 1). This structural characteristic may have important mechanistic implications. Specifically, reduced damage to the outer wall of Schlemm’s canal may contribute to preservation of collector channel ostia and attenuation of postoperative inflammation, both of which are essential for maintaining physiological aqueous outflow. These effects may, in turn, reduce the likelihood of transient postoperative intraocular pressure (IOP) spikes, which are commonly observed after trabecular surgery. However, the efficacy has not been adequately evaluated.

In this study, we retrospectively compared one-year surgical outcomes between 180° ab interno trabeculotomy combined with phacoemulsification using the Kahook Dual Blade (K group) and the T-hook (T group) in eyes with primary open-angle glaucoma (POAG).

## 2. Material and Methods

### 2.1. Participants

This retrospective study included patients with POAG who underwent 180° ab interno trabeculotomy combined with phacoemulsification at Kansai Medical University Hospital between June 2018 and September 2024 and were followed for at least 12 months postoperatively. Patients who did not complete at least 12 months of postoperative follow-up or had insufficient postoperative data were excluded from the analysis. Eyes with a history of additional glaucoma surgery, including laser procedures, or intraoperative complications during cataract surgery were excluded.

Patient demographic and clinical data, including age, sex, preoperative and postoperative intraocular pressure (IOP) at each follow-up visit, number of antiglaucoma medications, surgical procedure, and the presence and type of postoperative complications, were retrospectively extracted from medical records and included in the analysis. This study was approved by the Institutional Review Board of Kansai Medical University Hospital (Protocol No. 2023270, 5 July 2024). The requirement for written informed consent was waived owing to the retrospective nature of the study.

All patients underwent comprehensive ophthalmic examinations, including slit-lamp biomicroscopy, gonioscopy, and intraocular pressure measurements during the follow-up period.

### 2.2. Surgical Procedures

All included eyes underwent combined cataract surgery. All surgical procedures were performed under local anesthesia (sub-Tenon’s anesthesia using 2% lidocaine) by experienced glaucoma surgeons.

#### 2.2.1. Kahook Dual Blade-Assisted 180° Ab Interno Trabeculotomy Combined with Phacoemulsification

Two corneal side ports were created at the 2- and 9-o’clock positions using a 20-gauge V-lance. A dispersive ophthalmic viscosurgical device, Shellgan^®^ (purified sodium hyaluronate and chondroitin sulfate sodium; Santen Company, Osaka, Japan), was applied to protect the corneal endothelium during surgery. Additionally Purified sodium hyaluronate (purified sodium hyaluronate; Alcon, Tokyo, Japan) was injected into the anterior chamber to maintain anterior chamber depth. After completion of continuous curvilinear capsulorhexis, a clear corneal incision was made, followed by hydrodissection and hydrodelineation. Phacoemulsification and aspiration were performed to remove the lens nucleus and cortex, and an intraocular lens was implanted into the capsular bag.

The patient’s head and the operating microscope were tilted to allow visualization of the iridocorneal angle using an Ocular Swan-Jacob gonioprism (Ocular Instruments, Bellevue, WA, USA). Prior to insertion of the Kahook Dual Blade into Schlemm’s canal, Healon V^®^ (purified sodium hyaluronate; AMO Japan, Tokyo, Japan) was injected into the anterior chamber. The tip of the Kahook Dual Blade was then inserted into Schlemm’s canal through the trabecular meshwork and advanced circumferentially to excise approximately 180° of the nasal trabecular meshwork. The OVD was subsequently removed by irrigation and aspiration using a balanced salt solution at the completion of trabeculotomy. intraoperative blood reflux was irrigated and aspirated, and a subconjunctival injection of dexamethasone was administered at the end of the procedure.

#### 2.2.2. T-Hook-Assisted 180° Ab Interno Trabeculotomy Combined with Phacoemulsification

Two corneal side ports were created at the 2- and 9-o’clock positions using a 20-gauge V-lance. A dispersive ophthalmic viscosurgical device, Shellgan, was applied to protect the corneal endothelium during surgery. Additionally purified sodium hyaluronate was injected into the anterior chamber to maintain anterior chamber depth. Continuous curvilinear capsulorhexis, hydrodissection, hydrodelineation, phacoemulsification, cortical aspiration, and intraocular lens implantation into the capsular bag were performed in a standard fashion.

The iridocorneal angle was visualized using an Ahmed DVX surgical goniolens (Ocular Instruments, Bellevue, WA, USA). Prior to insertion of the T-hook into Schlemm’s canal, Healon V was injected into the anterior chamber. The tip of the T-hook was inserted into Schlemm’s canal via the trabecular meshwork and advanced circumferentially to incise approximately 180° of the nasal trabecular meshwork. The OVD was subsequently removed by irrigation and aspiration using a balanced salt solution at the completion of trabeculotomy. Intraoperative blood reflux was irrigated and aspirated, and a subconjunctival injection of dexamethasone was administered at the end of the procedure.

The iridocorneal angle was visualized using an Ocular Swan-Jacob gonioprism in the Kahook Dual Blade and an Ahmed DVX surgical goniolens in the T-hook. The choice of goniolens differed according to the structural characteristics of each device. Because the Kahook Dual Blade has a straight configuration between the shaft and tip, the use of a goniolens that fully covers the cornea, such as the Ahmed DVX lens, can hinder instrument maneuverability. Therefore, a Swan-Jacob gonioprism was used to allow more comfortable manipulation. In contrast, the T-hook has an angled tip relative to its shaft, enabling smooth handling even when using a cornea-covering goniolens such as the Ahmed DVX lens.

In both groups, at the end of the procedure, the absence of aqueous humor outflow from the wound was confirmed, and intraocular pressure was verified to be at least within the normal range by digital palpation. Postoperative management was performed according to standard clinical practice at our institution. Patients were instructed to maintain a postoperative head position such that the incision site was oriented upward until the morning of the first postoperative day. This approach was intended to prevent blood accumulation at the incision site and to facilitate its redistribution away from the treated angle. This postoperative instruction was applied consistently throughout the study period.

### 2.3. Outcome Measures

The primary outcome measures were:

(1) Changes in intraocular pressure (IOP) and mean IOP reduction rate,
(2) Changes in the number of antiglaucoma medications,
(3) Cumulative surgical success rates evaluated using Kaplan–Meier survival analysis, and
(4) The incidence of postoperative complications, including hyphema and transient IOP spikes.

IOP and the number of antiglaucoma medications were assessed preoperatively and at 1 week and 1, 3, 6, 9, and 12 months postoperatively. Baseline IOP was defined as the mean of the three most recent measurements obtained before trabeculotomy. Antiglaucoma medications included prostaglandin analogs, β-blockers, carbonic anhydrase inhibitors, α2-agonists, and ROCK inhibitors, pilocarpine, with each topical medication counted as one point. Combination therapies were counted as separate medications, whereas oral carbonic anhydrase inhibitors were counted as two points. Surgical failure in Kaplan–Meier survival analysis was defined as an IOP > 21 mmHg on two consecutive visits, a reduction in IOP of <20% from baseline on two consecutive visits, or the need for additional glaucoma surgery [12]. Postoperative complications included hyphema and transient IOP spikes. Hyphema was defined as ≥2 mm of layered blood in the anterior chamber within 1 week postoperatively confirmed by slit-lamp biomicroscopy. Transient IOP spikes were defined as an increase in IOP of ≥5 mmHg from baseline within 1 week after surgery.

## 3. Statistical Analysis

All statistical analyses were performed using JMP student Edition 19 software (SAS Institute Inc., Cary, NC, USA). Data are presented as median values with interquartile ranges unless otherwise specified.

Baseline demographic and clinical characteristics were compared between the K group and the T group. Age was expressed as mean ± standard deviation and compared using Welch’s *t*-test. Gender distribution was analyzed using the chi-square test. Preoperative IOP values were expressed as median with interquartile range and compared between groups using the Wilcoxon rank-sum test.

Because both eyes from the same patient were included in the analysis, mixed-effects models were used for comparative analyses to account for potential inter-eye correlation. Longitudinal changes in IOP and medication scores were analyzed using a linear mixed-effects model with patient ID as a random effect to account for inter-eye correlation. Comparisons of each postoperative time point with baseline were adjusted using Dunnett’s method.

Analysis of covariance (ANCOVA) was performed to compare the IOP reduction rate between groups while adjusting for baseline IOP.

Cumulative surgical success rates were evaluated using Kaplan–Meier survival analysis and compared between groups using the log-rank test.

The incidence of postoperative complications, including hyphema and transient IOP spikes, was compared between groups using Fisher’s exact test.

To identify factors associated with postoperative IOP spike, multivariable logistic regression analysis was performed. The presence of postoperative IOP spike was used as the dependent variable. Independent variables entered into the model included surgical device (Kahook Dual Blade vs. T-hook), number of preoperative glaucoma medications, baseline intraocular pressure (IOP), age, and sex. Odds ratios (ORs) with 95% confidence intervals (CIs) were calculated. Statistical significance was defined as *p* < 0.05.

In addition, propensity score matching analysis was performed as a sensitivity analysis to reduce baseline imbalance between groups. Propensity scores were estimated using a logistic regression model based on baseline variables including preoperative intraocular pressure and preoperative medication score. One-to-one nearest-neighbor matching without replacement was performed. Because of the limited sample size and the concern regarding substantial reduction in matched cases, a caliper was not applied. Covariate balance after matching was assessed using standardized mean differences. Baseline characteristics after matching were compared using the Wilcoxon rank-sum test, and the incidence of postoperative IOP spikes was compared using Fisher’s exact test.

All tests were two-sided, and *p* < 0.05 was considered statistically significant.

## 4. Results

A total of 61 eyes from 45 patients were included in the analysis, comprising 42 eyes (29 patients) in the K group and 19 eyes (16 patients) in the T group. Baseline clinical characteristics are summarized in Table 1. The male-to-female ratio was 15:27 in the K group and 6:13 in the T group, with no significant difference between groups (*p* = 0.7500). The mean age was 69.9 ± 7.3 years in the K group and 73.8 ± 9.6 years in the T group, with no significant intergroup difference (*p* = 0.1230). Baseline IOP was significantly higher in the K group than in the T group [18.7 (15.8–20.5) vs. 16.0 (14.0–18.0) mmHg, *p* = 0.0132]. Preoperative medication scores were not significantly different between the K group and the T group [4 (2–4) vs. 4 (3–5), *p* = 0.0580] (Table 1).

**Figure 2:**
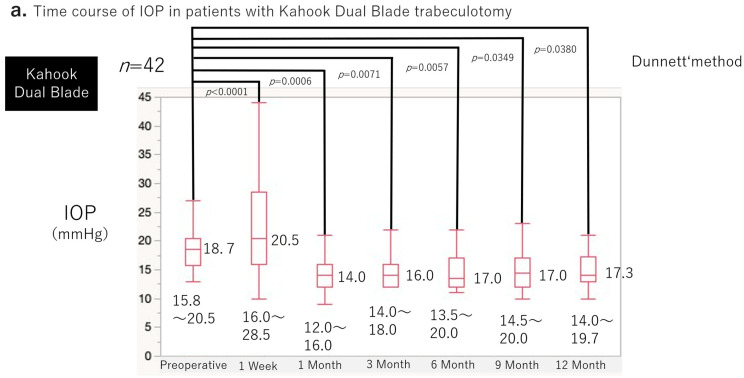

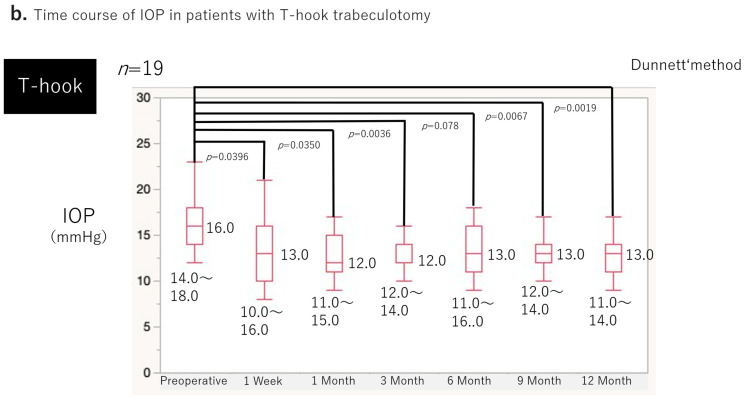
Time course of intraocular pressure following 180° ab interno trabeculotomy. (**a**) Kahook Dual Blade group (*n* = 42 eyes). (**b**) T-hook group (*n* = 19 eyes).

Figure 2a,b show the preoperative and postoperative IOP changes in both groups. A transient increase in IOP at postoperative week 1 was observed in the K group (*p* < 0.0001). From 1 month onward, both groups demonstrated significant reductions in IOP compared with baseline at all postoperative time points (*p* < 0.05). At 12 months, the mean IOP reduction rate was 17.2% in the K group and 20.1% in the T group, both showing significant decreases from baseline (*p* < 0.05 for each group). After adjusting for baseline IOP using analysis of covariance, no significant difference in IOP reduction rate was observed between groups (*p* = 0.0720, ANCOVA).

Intraocular pressure (IOP) values at each time point are presented as median with interquartile range. The median preoperative IOP was 18.7 mmHg (15.8–20.5). IOP increased transiently at postoperative week 1 to 20.5 mmHg (16.0–28.5; *p* = 0.0427 vs. baseline). From 1 month onward, IOP was significantly reduced compared with baseline at all follow-up time points (all *p* < 0.0001).

The median preoperative IOP was 16.0 mmHg (14.0–18.0). IOP did not demonstrate an early postoperative increase and showed a decreasing trend as early as week 1 (median 13.0 mmHg [10.0–16.0]; *p* = 0.0509). Significant IOP reductions were observed from 1 month through 12 months (all *p* < 0.05).

**Figure 3:**
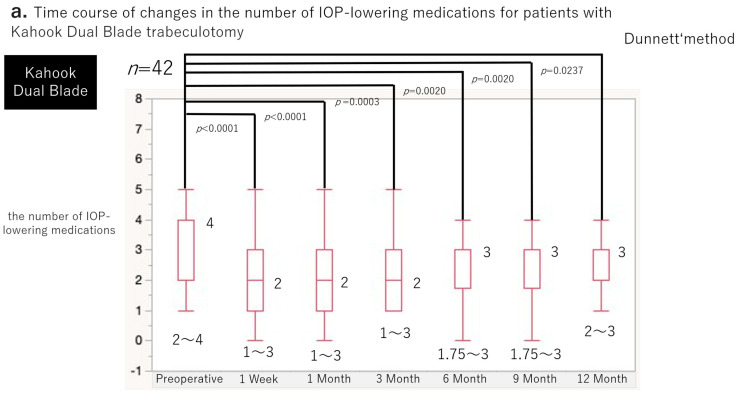

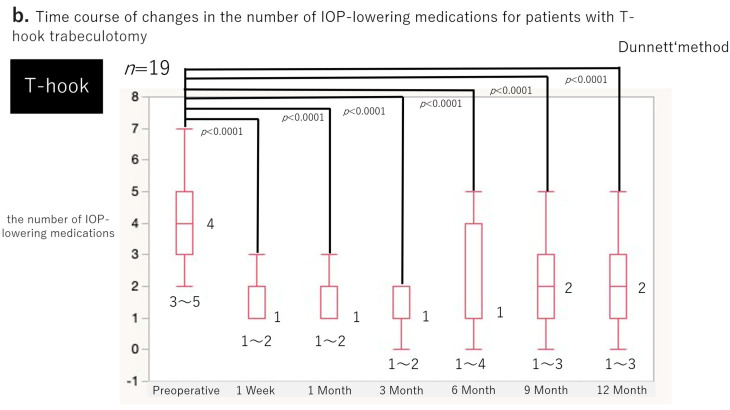
Time course of changes in the number of Intraocular pressure (IOP)-lowering medications following 180° ab interno trabeculotomy. (**a**) Kahook Dual Blade group (*n* = 42 eyes). (**b**) T-hook group (*n* = 19 eyes).

Changes in the number of antiglaucoma medications are shown in Figure 3a,b. Both groups demonstrated significant reductions in medication scores at all postoperative time points compared with baseline (*p* < 0.05).

Data are presented as medians with interquartile ranges.

In the K group, the median preoperative medication score was 4 (2–4). The score decreased significantly to 2 (1–3) at postoperative week 1 (*p* = 0.0060) and remained significantly lower than baseline at 1 month (2 [1–3]; *p* < 0.0001), 3 months (2 [1–3]; *p* = 0.0020), 6 months (3 [1.75–3]; *p* = 0.0080), 9 months (3 [1.75–3]; *p* = 0.0080), and 12 months (3 [2–3]; *p* = 0.0110).

In the T group, the median preoperative medication score was 4 (3–5). The score decreased significantly to 1 (1–2) at postoperative week 1 (*p* < 0.0001) and remained significantly lower than baseline at 1 month (1 [1–2]; *p* < 0.0001), 3 months (1 [1–2]; *p* < 0.0001), 6 months (1 [1–4]; *p* = 0.0003), 9 months (2 [1–3]; *p* = 0.0004), and 12 months (2 [1–3]; *p* = 0.0003).

**Figure 4:**
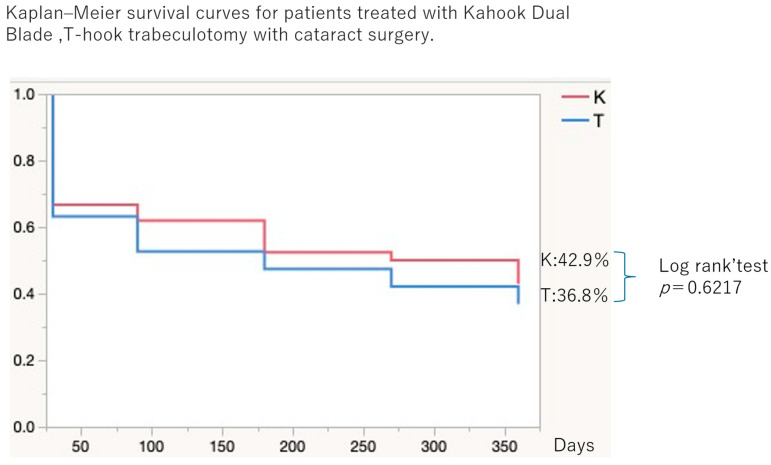
Kaplan–Meier survival curves for cumulative surgical success following 180° ab interno trabeculotomy combined with phacoemulsification.

Figure 4 presents the cumulative surgical success rates based on Kaplan–Meier survival analysis. At 12 months, the cumulative probability of success was 42.9% in the K group and 36.8% in the T group, with no significant difference between groups (*p* = 0.6217).

Kaplan–Meier survival curves illustrate cumulative surgical success in the Kahook Dual Blade group (K group; *n* = 42 eyes) and the T-hook group (T group; *n* = 19 eyes) over a 12-month follow-up period. At 12 months, the cumulative probability of surgical success was 42.9% in the K group and 36.8% in the T group. Survival distributions were compared using the log-rank test, which demonstrated no statistically significant difference between groups (*p* = 0.6217).

Early postoperative complications are summarized in Table 2. The incidence of hyphema was 9.5% in the K group and 5.3% in the T group, with no significant difference between groups (*p* = 1.00, Fisher’s exact test). In contrast, transient IOP spikes occurred significantly more frequently in the K group (40.5%) than in the T group (5.3%) (*p* = 0.0057, Fisher’s exact test).

To identify factors associated with postoperative IOP spike, multivariable logistic regression analysis was performed (Table 3). Surgical device (OR 29.73, 95% CI 3.291–893.5, *p* = 0.0010), number of preoperative glaucoma medications (OR 2.590, 95% CI 1.377–5.916, *p* = 0.0019), and baseline intraocular pressure (OR 1.280, 95% CI 1.035–1.646, *p* = 0.0215) were independently associated with postoperative IOP spike. Age (*p* = 0.4707) and sex (*p* = 0.5797) were not significantly associated with IOP spike.

In addition, propensity score matching analysis was performed using preoperative IOP and preoperative medication score to reduce baseline imbalance between groups. After one-to-one nearest-neighbor matching, baseline imbalance between groups was reduced, with a standardized mean difference of 0.148 for preoperative IOP and 0.060 for preoperative medication score. No significant differences were observed between groups in preoperative IOP (*p* = 0.7686) or preoperative medication score (*p* = 1.0000). In the matched cohort, transient postoperative IOP spikes remained significantly more frequent in the K group than in the T group (*p* = 0.0188, Fisher’s exact test) (Table 4).

## 5. Discussion

In this study, we compared the 1-year outcomes of 180° ab interno trabeculotomy combined with phacoemulsification using the T-hook and the Kahook Dual Blade in eyes with primary open-angle glaucoma (POAG). Both groups demonstrated significant reductions in intraocular pressure (IOP) and antiglaucoma medication use after surgery, with no significant differences between groups in IOP reduction, IOP reduction rate, medication reduction, or cumulative surgical success. However, a notable difference was observed in the incidence of transient postoperative IOP spikes, which occurred significantly more frequently in the K group than in the T group (40.5% vs. 5.3%). This difference remained significant even after propensity score matching analysis. These findings suggest that, although the two procedures provide comparable IOP-lowering efficacy, differences may exist in early postoperative outcomes, particularly transient postoperative IOP spikes.

Although the present study specifically compared the T-hook with the Kahook Dual Blade, several previous comparative studies evaluating Kahook Dual Blade and other trabecular hook-based procedures, including Tanito microhook trabeculotomy (TMH), provide important methodological context for interpreting the current findings. Previous retrospective comparative studies generally demonstrated broadly comparable long-term IOP-lowering efficacy among trabeculotomy-based MIGS devices, including Kahook Dual Blade, TMH, and T-hook procedures [13,14,15,16,17,18]. Tanito et al. also reported favorable midterm surgical outcomes of TMH in a large case series [13]. Furthermore, Chihara et al. compared multiple canal-opening procedures, including Kahook Dual Blade, TMH, and T-hook, and reported broadly comparable long-term outcomes among sectoral trabeculotomy devices [14]. Propensity score-adjusted comparative studies by Nakagawa et al. and Okada et al. demonstrated no significant differences in long-term postoperative IOP control or surgical success between microhook-based trabeculotomy procedures and Kahook Dual Blade goniotomy [15,16]. Comparative studies involving other trabecular MIGS devices, including iStent and Kahook Dual Blade, have likewise demonstrated generally similar long-term efficacy despite differences in surgical mechanism and incision architecture [17,18]. In addition, recent systematic reviews and meta-analyses of trabecular MIGS procedures have concluded that no clear superiority of a specific device has been consistently established with respect to long-term IOP-lowering efficacy [1,4]. Collectively, these studies suggest that long-term surgical efficacy may be generally comparable among trabeculotomy-based MIGS procedures, even after adjustment for baseline differences.

In contrast, reports regarding transient postoperative IOP spikes have been less consistent. Several comparative studies, including propensity score-adjusted analyses, reported no significant differences in postoperative IOP spike incidence among devices [15,16]. However, Chihara et al. observed that transient postoperative IOP elevation tended to occur less frequently in the T-hook cohort during the early postoperative period [14]. Comparative studies between Kahook Dual Blade and iStent procedures have also suggested that postoperative hyphema and blood reflux may occur more frequently in canal-opening procedures involving trabecular incision or excision [17,18]. Previous studies have further suggested that postoperative hyphema and retained blood components may contribute to transient postoperative IOP elevation by increasing resistance within the aqueous outflow pathway [12,19,20,21]. Collectively, these findings suggest that, although long-term IOP-lowering efficacy may be broadly comparable among trabeculotomy devices, device-specific surgical characteristics may influence early postoperative IOP behavior.

Transient postoperative IOP spikes are clinically relevant because acute IOP elevations may adversely affect already compromised glaucomatous eyes and often require additional postoperative management [21]. Several mechanisms have been proposed for postoperative IOP elevation following ab interno trabeculotomy, including delayed hyphema absorption, obstruction of aqueous outflow by degenerated erythrocytes or thrombus formation, and postoperative inflammatory changes [19,20,21]. In the present study, one possible explanation for the lower incidence of postoperative IOP spikes in the T-hook group is that the structural design of the T-hook may result in less tissue trauma to the trabecular meshwork and surrounding tissues, thereby reducing postoperative bleeding and inflammatory obstruction of aqueous outflow. Furthermore, compared with sharper excisional devices such as the Kahook Dual Blade, the relatively blunt or rounded tip design of hook-based devices, including the T-hook and Tanito microhook, may reduce damage to the outer wall of Schlemm’s canal and collector channel ostia. However, because inflammatory biomarkers and postoperative blood retention were not directly evaluated in this study, the underlying mechanisms remain speculative.

Chihara et al. [11] previously reported similar rates of postoperative IOP spikes between the T-hook and Kahook Dual Blade groups (41.2% vs. 47.5%), whereas the present study demonstrated a significantly lower incidence in the T-hook group (5.3% vs. 40.5%). One possible explanation for this discrepancy is the difference in patient selection criteria. Chihara et al. limited their analysis to eyes with a preoperative IOP ≥ 21 mmHg, whereas the present study included a broader range of patients with POAG requiring trabeculotomy. Although this broader inclusion criterion may have introduced greater heterogeneity, it may also better reflect real-world clinical practice. Furthermore, additional propensity score matching analysis demonstrated that the difference in postoperative IOP spike incidence remained significant even after adjustment for baseline imbalance, supporting the robustness of the observed association. Therefore, the findings of the present study may provide clinically relevant information regarding the association between device type and early postoperative IOP behavior in routine clinical settings.

Several limitations of this study should be acknowledged. This was a retrospective, non-randomized study with partially sequential cohorts, as the T-hook was introduced later than the Kahook Dual Blade. This may have introduced selection bias and confounding related to differences in surgical experience, learning-curve effects, and patient selection. In addition, baseline intraocular pressure was higher in the K group, which may have influenced postoperative outcomes despite statistical adjustment. Although additional propensity score matching analysis was performed to reduce baseline imbalance, residual confounding inherent to the retrospective study design cannot be fully excluded, and therefore device-specific effects cannot be completely isolated. Furthermore, the relatively small sample size, particularly in the T-hook group, together with the 12-month follow-up period, may have limited statistical power and the ability to assess long-term durability. Residual confounding cannot be excluded, including baseline imbalance and potential inter-eye correlation. In addition, the definition of IOP spike used in this study (≥5 mmHg increase within 1 week) may capture relatively mild fluctuations compared with stricter criteria used in prior studies, potentially leading to a higher reported incidence. The use of a more stringent definition would likely reduce the absolute incidence of IOP spikes and may attenuate the observed difference between groups, although the overall trend might remain unchanged. Similarly, the success criteria requiring both an IOP ≤ 21 mmHg and a ≥20% reduction from baseline on two consecutive visits may have underestimated Kaplan–Meier survival rates, particularly in eyes with lower baseline IOP. Furthermore, the relatively low success rates observed at 12 months, which were below the proposed minimal clinically important difference (MCID) threshold of 50%, suggest that outcomes may further decline with longer follow-up, indicating a potential limitation in long-term durability. Finally, although propensity score matching was additionally performed, the relatively small sample size limited the robustness of the matching process, and the results should therefore be interpreted with caution.

Despite these limitations, both procedures demonstrated comparable intraocular pressure-lowering efficacy, while T-hook-assisted trabeculotomy was associated with fewer transient postoperative IOP spikes during the early postoperative period in this cohort.

## 6. Conclusions

While both procedures demonstrated comparable intraocular pressure-lowering efficacy, T-hook-assisted trabeculotomy was associated with fewer transient postoperative IOP spikes during the early postoperative period in this cohort.

## Figures and Tables

**Table 1:** Baseline patient characteristics.

	Eyes	Mean Age	Gender Ratio (M:F)
Kahook Dual Blade	42	69.9 ± 7.3	15:27
T-hook	19	73.8 ± 9.6	6:13
*p*-value		0.1230 (Welch’s *t*-test)	0.7500 (Chi-square test)
		Preoperative intraocular pressure	
Kahook Dual Blade		18.7 (15.8–20.5)	
T-hook		16.0 (14.0–18.0)	
*p*-value		0.0132 (Wilcoxon rank-sum test)	
		the number of antiglaucoma medications	
Kahook Dual Blade		4 (2–4)	
T-hook		4 (3–5)	
*p*-value		0.0580 (Wilcoxon rank-sum test)	

**Table 2:** Early postoperative complications.

	The Rate of Hyphema	The Rate of IOP Spike
Kahook Dual Blade	4/42 (9.5%)	17/42 (40.5%)
T-hook	1/19 (5.3%)	1/19 (5.3%)
*p* value	1.00 (Fisher’s exact test)	0.0057 (Fisher’s exact test)

**Table 3:** Multivariable Logistic Regression Analysis for IOP Spike.

Comparison	Odds Ratio	95%CL Lower	95%CL Upper	*p* Value
K vs. T	29.7336	3.2906	893.5069	0.0010
Variable	Odds Ratio	95%CL Lower	95%CL Upper	*p* value
Preoperative Glaucoma medications	2.5898	1.3772	5.9158	0.0019
Preoperative IOP	1.2794	1.0352	1.6458	0.0215
Age	0.9627	0.8629	1.0684	0.4707
Sex	0.6417	0.1261	3.1620	0.5797

**Table 4:** Preoperative and Postoperative IOP, Medication Score, IOP Reduction Rate, and Postoperative Complications After Propensity Score Matching.

	T-Hook (*n* = 19)	After Matching Kahook Dual Blade (*n* = 19)	*p* Value	SMD
Pre operative IOP	16.0 ± 2.9	16.4 ± 2.5	0.7686	0.148
12-Month Postoperative IOP	12.8 ± 2.3	14.2 ± 2.6	0.1030	0.570
IOP reduction rate	18.6 ± 16.7(%)	12.3 ± 14.9(%)	0.1080	0.400
Pre operative Medication score	4.05 ± 1.07	4.0 ± 0.58	1.0000	0.060
12-Month Postoperative Medication score	2.00 ± 1.49	2.47 ± 0.96	0.2629	0.380
The Rate of IOP Spike	1/19(5.3%)	8/19(42.3%)	0.0188	0.960

## Data Availability

The authors confirm that the data supporting the findings of this study are available within the article.
